# Tumor-derived soluble CD155 inhibits DNAM-1–mediated antitumor activity of natural killer cells

**DOI:** 10.1084/jem.20191290

**Published:** 2020-02-10

**Authors:** Genki Okumura, Akiko Iguchi-Manaka, Rikito Murata, Yumi Yamashita-Kanemaru, Akira Shibuya, Kazuko Shibuya

**Affiliations:** 1 Department of Immunology, University of Tsukuba, Tsukuba, Ibaraki, Japan; 2 Breast and Endocrine Surgery, Faculty of Medicine, University of Tsukuba, Tsukuba, Ibaraki, Japan; 3 Doctoral Program of Biomedical Sciences, Comprehensive Human Sciences, University of Tsukuba, Tsukuba, Ibaraki, Japan; 4 PhD Program in Human Biology, School of Integrative and Global Majors, University of Tsukuba, Tsukuba, Ibaraki, Japan; 5 Life Science Center for Survival Dynamics, Tsukuba Advanced Research Alliance, University of Tsukuba, Tsukuba, Ibaraki, Japan; 6 R&D Center for Innovative Drug Discovery, University of Tsukuba, Tsukuba, Ibaraki, Japan

## Abstract

CD155 is a ligand for DNAM-1, TIGIT, and CD96 and is involved in tumor immune responses. Unlike mouse cells, human cells express both membranous CD155 and soluble CD155 (sCD155) encoded by splicing isoforms of *CD155*. However, the role of sCD155 in tumor immunity remains unclear. Here, we show that, after intravenous injection with sCD155-producing B16/BL6 melanoma, the numbers of tumor colonies in wild-type (WT), TIGIT knock-out (KO), or CD96 KO mice, but not DNAM-1 KO mice, were greater than after injection with parental B16/BL6 melanoma. NK cell depletion canceled the difference in the numbers of tumor colonies in WT mice. In vitro assays showed that sCD155 interfered with DNAM-1–mediated NK cell degranulation. In addition, DNAM-1 had greater affinity than TIGIT and CD96 for sCD155, suggesting that sCD155 bound preferentially to DNAM-1. Together, these results demonstrate that sCD155 inhibits DNAM-1–mediated cytotoxic activity of NK cells, thus promoting the lung colonization of B16/BL6 melanoma.

## Teaser

This report demonstrated that soluble CD155 interferes with the DNAM-1–mediated antitumor immunity mediated by NK cells and promotes lung colonization of B16/BL6 melanoma cells.

## Introduction

Cluster of differentiation 155 (CD155) was originally identified as a poliovirus receptor (Mendelsohn et al., 1989). It is also known as an adhesion molecule termed nectin-like protein-5, belonging to the immunoglobulin superfamily with three extracellular immunoglobulin-like domains (Takai et al., 2008). CD155 is expressed on both hematopoietic and nonhematopoietic cells. Importantly, it is also a stress-inducible protein, the production of which is up-regulated on transformed cells (Bevelacqua et al., 2012; Masson et al., 2001; Nakai et al., 2010; Nishiwada et al., 2015). CD155 plays key roles in cell migration (Ikeda et al., 2004; Sloan et al., 2004) and proliferation (Kakunaga et al., 2004) through interaction with other adhesion molecules such as integrin αvβ3 and the extracellular matrix protein vitronectin (Takai et al., 2008).

Accumulating evidence has demonstrated that CD155 is also a ligand for the activating receptor DNAM-1 (DNAX accessory molecule 1, CD226; Bottino et al., 2003; Shibuya et al., 1996; Tahara-Hanaoka et al., 2004) and the inhibitory receptors TIGIT (T cell immunoglobulin and immunoreceptor tyrosine-based inhibitory motif domain; Yu et al., 2009) and CD96 (also known as TACTILE: T cell activation, increased late expression; Fuchs et al., 2004; Wang et al., 1992). These receptors are expressed on resting or activated T cells, or both, and natural killer (NK) cells. Interaction of DNAM-1 expressed on CD8^+^ T cells and NK cells with CD155 expressed on target cells mediates an activation signal for cytotoxicity and cytokine production in T cells and NK cells (Iguchi-Manaka et al., 2008; Tahara-Hanaoka et al., 2006). In contrast, TIGIT and CD96 contain immunoreceptor tyrosine-based inhibitory motif in their cytoplasmic portions and mediate an inhibitory signal in NK cells upon binding to CD155 on target cells (Dougall et al., 2017). CD155-mediated immune regulation via interaction with these receptors is thus complicated, and it is incompletely understood.

Interestingly, unlike in mice, in humans CD155 is expressed not only as a membrane-bound protein encoded by *CD155α* and *CD155δ* but also as soluble forms lacking the transmembrane region and encoded by the alternative splicing isoforms *CD155β* and *CD155γ* (Baury et al., 2003). Although soluble CD155 (sCD155) contributes to the neutralization of poliovirus infectivity in vitro (Baury et al., 2003), it remains undetermined how it is involved in tumor immune responses mediated by DNAM-1, TIGIT, and CD96. In our previous study, we found that serum levels of sCD155 were higher in patients with various types of cancer than in healthy people and that patients with advanced-stage cancers had higher levels of sCD155 than did those with early-stage cancers (Iguchi-Manaka et al., 2016). In addition, serum levels of sCD155 were positively correlated with tumor size (Iguchi-Manaka et al., 2016). Although it remains unclear whether increased sCD155 production is a cause of tumor development, sCD155 may modulate tumor immune responses through interaction with any, or all, of DNAM-1, TIGIT, and CD96 on T cells and NK cells. Here, we investigated the role of sCD155 in tumor immunity by using the B16/BL6 lung colonization model in mice. We demonstrated that sCD155 promotes lung colonization of B16/BL6 cells by suppressing DNAM-1–mediated NK cell function.

## Results and discussion

### sCD155 suppresses NK cell function against lung colonization of B16/BL6 melanoma

**Figure S1.**
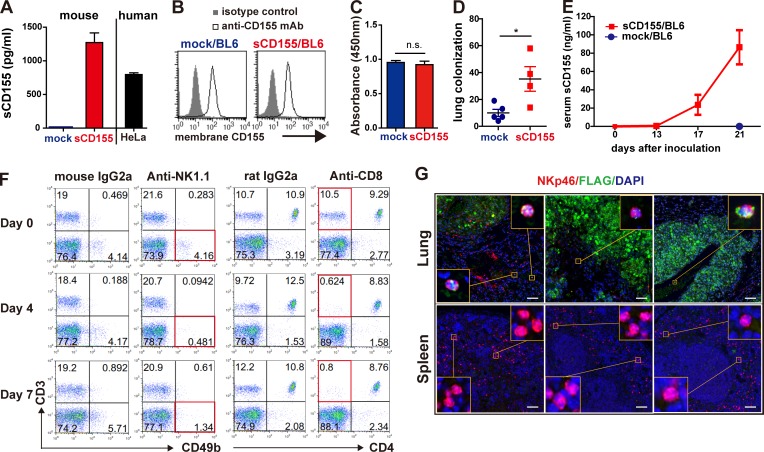
**Characterization of generated sCD155/BL6 and mock/BL6, and serum levels of sCD155.** **(A)** Concentrations of mouse and human sCD155 in the culture supernatant of sCD155/BL6 (*n* = 3), mock/BL6 (*n* = 3), and HeLa (*n* = 3) were analyzed 24 h after the start of the culture by CBA assay and ELISA, respectively. **(B)** Expression of membrane-bound CD155 on sCD155/BL6 and mock/BL6 was analyzed by using flow cytometry. **(C)** sCD155/BL6 (*n* = 3) and mock/BL6 (*n* = 3) were cultured (1.0 × 10^5^ cells/well) in 96-well flat plates for 24 h, and then BrdU reagent was added to the cultures. BrdU incorporation was measured after culture for 12 h. **(D)** C57BL/6 WT mice were intravenously injected with different clones of sCD155/BL6 (*n* = 4) and mock/BL6 (*n* = 5) from those used in Fig. 1. Colony numbers in the lung were counted on day 17. **(E)** C57BL/6 WT mice were intravenously injected with sCD155/BL6 (*n* = 5) or mock/BL6 (*n* = 5) used in Fig. 1 and Fig. 2, and analyzed for serum levels of sCD155 on days 0, 13, 17, and 21. **(F)** C57BL/6 WT mice were treated with mouse IgG2a, anti-NK1.1 antibody, rat IgG2a, or anti-CD8 antibody. Peripheral blood mononuclear cells on days 0, 4, and 7 were stained with antibodies against CD3, CD49b, and/or CD4. **(G)** C57BL/6 WT mice were intravenously injected with sCD155/BL6 or mock/BL6. Paraffin sections of lungs with colonized tumor and spleen on day 17 were stained as described in Fig. 1 F. Scale bars, 50 µm. Error bars indicate SD. Results were analyzed by using Student’s *t* test. For all analyses: *, P < 0.05; n.s., not significant.

**Figure 1.**
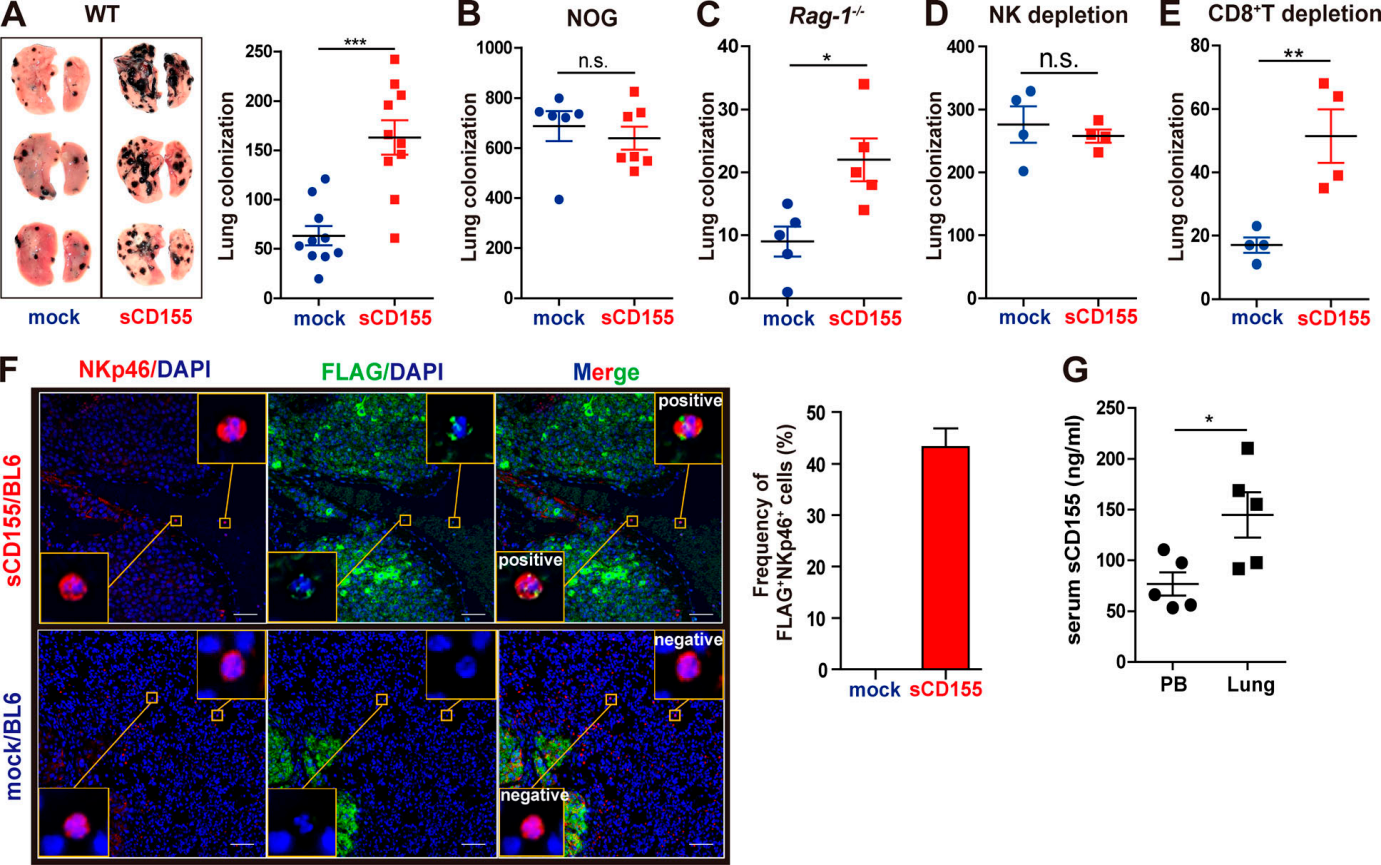
**sCD155 suppresses NK cell function against lung colonization of B16/BL6 melanoma.** **(A–C)** C57BL/6 WT (*n* = 10 in each group), NOG (*n* = 7 and 6 for sCD155/BL6 and mock/BL6, respectively), or *Rag-1*^−/−^ (*n* = 5 in each group) mice were intravenously inoculated with sCD155/BL6 or mock/BL6. Lung metastases were quantified by counting metastatic foci on the lung surface on day 17 (A and C) and day 12 (B). Representative images of lungs with metastases are shown (A). Results of three (A) or two (B and C) independent experiments were pooled for statistical analyses. **(D and E)** NK cells and CD8^+^ T cells were depleted from C57BL/6 WT mice by intraperitoneal administration of anti-NK1.1 mAb or anti-CD8 mAb. These mice (*n* = 4 in each group) were intravenously inoculated with sCD155/BL6 or mock/BL6 on day 0. Lung metastases were quantified by counting metastatic foci on the lung surface on day 17. Data are representative of two independent experiments. **(F)** C57BL/6 mice were intravenously inoculated with sCD155/BL6 or mock/BL6. Paraffin sections of lung with metastases were stained for FLAG (sCD155-FLAG; green) and NKp46 (red) by using the Opal multiplex immunofluorescence staining with DAPI (blue). Note that mock/BL6 also express Flag. Slides were then observed in a Mantra Quantitative Pathology Workstation. Representative FLAG-positive and -negative images are shown in the yellow squares. Scale bars, 50 µm. For the bar graph, the frequencies (%) of FLAG^+^ NKp46^+^ cells among NKp46^+^ cells in three lungs each for the sCD155/BL6 and mock/BL6 treatments were quantified. **(G)** Serum levels of sCD155 in peripheral blood (PB) and pulmonary vein of mice were analyzed on day 17 after injection of sCD155/BL6 (*n* = 5 in each group). Results were analyzed by using Student’s *t* test. Error bars indicate SD. For all analyses: *, P < 0.05; **, P < 0.01; ***, P < 0.001; n.s., not significant.

Unlike in humans, sCD155 is not expressed in mice. Therefore, to examine the role of sCD155 in tumor immunity, we established a transfectant of B16/BL6 mouse melanoma, which expressed the extracellular domain of mouse sCD155 tagged with FLAG protein at the C terminus (sCD155/BL6), and a mock transfectant (mock/BL6). The sCD155/BL6 produced a comparable amount of sCD155 to that naturally produced by the human cancer cell line HeLa (Fig. S1 A). The expression level of membrane CD155 and the in vitro cell proliferation were also comparable between these transfectants (Fig. S1, B and C). We then created a lung tumor colonization model by intravenous injection of these transfectants into WT mice. On day 17 after injection of the transfectant, mice that had received sCD155/BL6 showed significantly augmented tumor colonization in the lung compared with those that had received mock/BL6 (Fig. 1 A), suggesting that tumor-derived sCD155 promotes lung tumor colonization of B16/BL6. We observed similar results when we used different clones of sCD155/BL6 and mock/BL6 (Fig. S1 D). We also found that serum levels of sCD155 on days 17–21 after injection of sCD155/BL6 were comparable to those in human cancer patients that were reported previously (Iguchi-Manaka et al., 2016; Fig. S1 E), suggesting that this tumor model in mice can be applied to the study of the role of sCD155 in tumor immunity in humans. When we injected NOG mice intravenously with sCD155/BL6 or mock/BL6, the colony numbers of both sCD155/BL6 and mock/BL6 in the lung were higher compared with WT mice and comparable between the two groups on day 12 after the injection (Fig. 1 B). In contrast, *Rag1*^−/−^ mice that received sCD155/BL6 had significantly more colonies by day 17 than those that received mock/BL6 (Fig. 1 C). NOG mice lack NK cells as well as T and B lymphocytes, whereas *Rag1*^−/−^ mice exhibit a deficiency of T and B lymphocytes, but not NK cells. These results therefore suggested that sCD155 suppressed NK cell function in suppression of lung tumor colonization of B16/BL6.

NK cells are major effector cells involved in antitumor immunity against experimental lung tumor colonization of B16/BL6 melanoma (Cuff et al., 2010), suggesting that sCD155 suppressed NK cell function in this model. To examine this hypothesis, we injected WT mice with sCD155/BL6 or mock/BL6 after depletion of NK cells by intraperitoneal administration of anti-NK1.1 mAb (Fig. S1 F). As expected, lung tumor colonization was increased in both mice that had received sCD155/BL6 and those that had received mock/BL6 after depletion of NK cells; notably, the numbers of colonies were comparable between the two groups (Fig. 1 D). In contrast, the number of tumor colonies was significantly greater in sCD155/BL6-injected WT mice than in mock/BL6-injected WT mice after depletion of CD8^+^ T cells by anti-CD8 mAb (Fig. 1 E and Fig. S1 F), although CD8^+^ T cell depletion decreased the tumor colony number in mice given sCD155/BL6 and those given mock/BL6. These findings are consistent with a previous report that NK cell activation is up-regulated in the absence of CD8^+^ T cells (Cuff et al., 2010).

To examine whether sCD155 interacts with tumor-infiltrating resident NK cells in the lung, we performed fluorescent multiplex immunohistochemistry of the lungs of mice that had received sCD155/BL6 or mock/BL6; we used anti-FLAG and anti-NKp46 mAbs to detect FLAG-tagged sCD155 and NK cells, respectively. The signals for FLAG-tagged sCD155 were merged with >40% of the NK cell signals in the lungs of mice that had received sCD155/BL6 (Fig. 1 F and Fig. S1 G). In a sharp contrast, the merged signals were not observed in the lungs of mice that had received mock/BL6 (Fig. 1 F). However, we did not observe the merged signal in NK cells in the spleen of mice that had received sCD155/BL6 (Fig. S1 G). Importantly, serum levels of sCD155 in the blood of the pulmonary vein of the lung colonized with sCD155/BL6 were significantly higher than those in the peripheral blood (Fig. 1 G). Together, these results suggested that the higher concentration of sCD155 affected NK cell function preferentially in the tumor microenvironment in the lung and exacerbated lung colonization of B16/BL6 melanoma.

### sCD155 interferes with DNAM-1–mediated immunity against lung colonization of B16/BL6 melanoma

**Figure 2.**
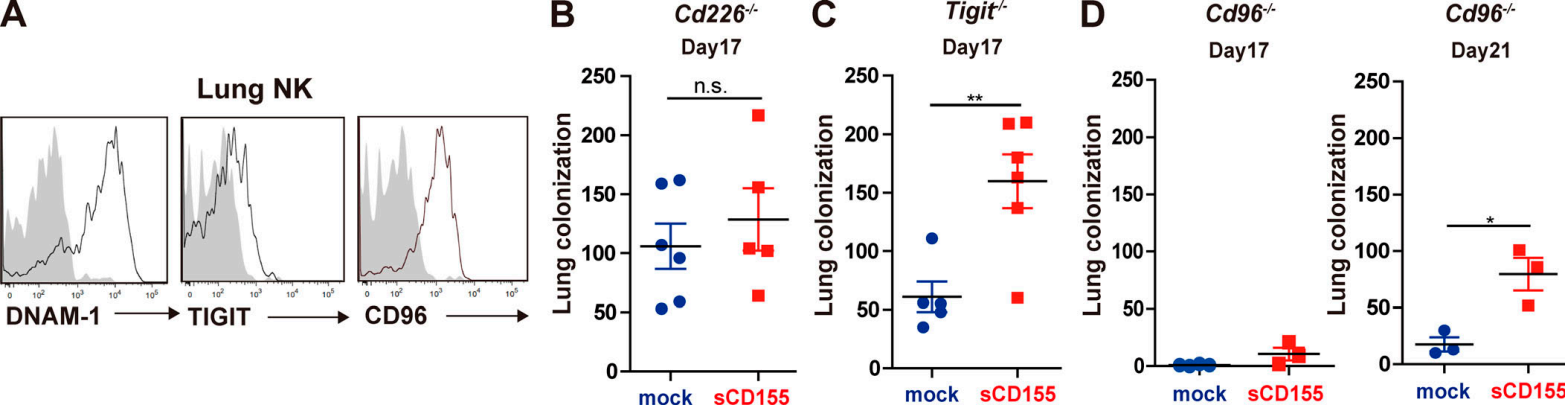
**sCD155 interferes with DNAM-1–mediated immunity against lung metastasis of B16/BL6 melanoma.** **(A)** C57BL/6 mice were intravenously inoculated with B16/BL6. On day 17, lung metastatic foci were resected under a stereo microscope. The resected tissues were mashed and passed through a mesh to prepare a single-cell suspension. Expression of DNAM-1, TIGIT, and CD96 on tumor-infiltrating NK cells was analyzed by using flow cytometry. **(B–D)** C57BL/6 *Cd226^−/−^* (*n* = 6 and 5 for sCD155/BL6 and mock/BL6, respectively), *Tigit^−/−^* (*n* = 6 and 5 for sCD155/BL6 and mock/BL6, respectively), and *Cd96^−/−^* (*n* = 3 in each group) mice were intravenously inoculated with sCD155/BL6 (sCD155) or mock/BL6 (mock). WT control that were performed in parallel are shown in Fig 1 A. Lung metastases were quantified by counting metastatic foci on the lung surface on day 17 (B–D, left). In the case of *Cd96*^−/−^ mice, lung metastases were also counted on day 21 (D, right). Results of two independent experiments were pooled for statistical analysis (B and C). Data are representative of two independent experiments (D). Error bars indicate SD. Results were analyzed by using Student’s *t* test. For all analyses: *, P < 0.05; **, P < 0.01; n.s., not significant.

DNAM-1, TIGIT, and CD96 are receptor ligands for CD155 (Bottino et al., 2003; Fuchs et al., 2004; Tahara-Hanaoka et al., 2004; Yu et al., 2009). We examined the expression of these CD155 ligands on resident NK cells in the lung. Whereas DNAM-1 and CD96 were highly expressed on lung NK cells, these cells expressed only small amounts of TIGIT (Fig. 2 A). Therefore, sCD155 was likely bound to either DNAM-1 or CD96 on NK cells, and this binding might have resulted in the suppression of NK cell activity. To find a functional NK cell receptor for sCD155 in the lung tumor colonization model, we intravenously injected *Cd226*^−/−^, *Tigit^–^*^/–^, or *Cd96*^−/−^ mice, as well as WT control mice (Fig. 1 A), with sCD155/BL6 or mock/BL6. By day 17, the number of tumor colonies in *Cd226*^−/−^ mice given mock/BL6 had increased to a level comparable to that in those given sCD155/BL6 (Fig. 2 B). In contrast, *Tigit*^−/−^ and *Cd96*^−/−^ mice given sCD155/BL6 still had significantly more tumor colonies than did those given mock/BL6 (Fig. 2, C and D), suggesting that impairment of NK cell–mediated antitumor immunity by sCD155 was dependent on DNAM-1, but not on TIGIT or CD96. These results thus suggested that sCD155 bound preferentially to DNAM-1 and interfered with DNAM-1–mediated activation of NK cells. We also observed that *Cd96*^−/−^ mice injected with either sCD155/BL6 or mock/BL6 had fewer colonies than did WT mice (Fig. 1 A and Fig. 2 D). In contrast, *Tigit*^−/−^ mice treated with either sCD155/BL6 or mock/BL6 showed no change in the number of tumor colonies in the lung compared with WT mice (Fig. 1 A and Fig. 2 C). Although both CD96 and TIGIT negatively regulate NK cell activation (Dougall et al., 2017), only CD96 suppressed NK cell–mediated tumor immunity against colonization of both sCD155/BL6 and mock/BL6 independently of sCD155, probably due to high expression of CD96, but not TIGIT, on lung NK cells (Fig. 2 A). Taken together, these results suggested that DNAM-1 is a functional NK cell receptor for sCD155 in this tumor colonization model.

### sCD155 suppresses DNAM-1–mediated degranulation activity of NK cells.

**Figure 3.**
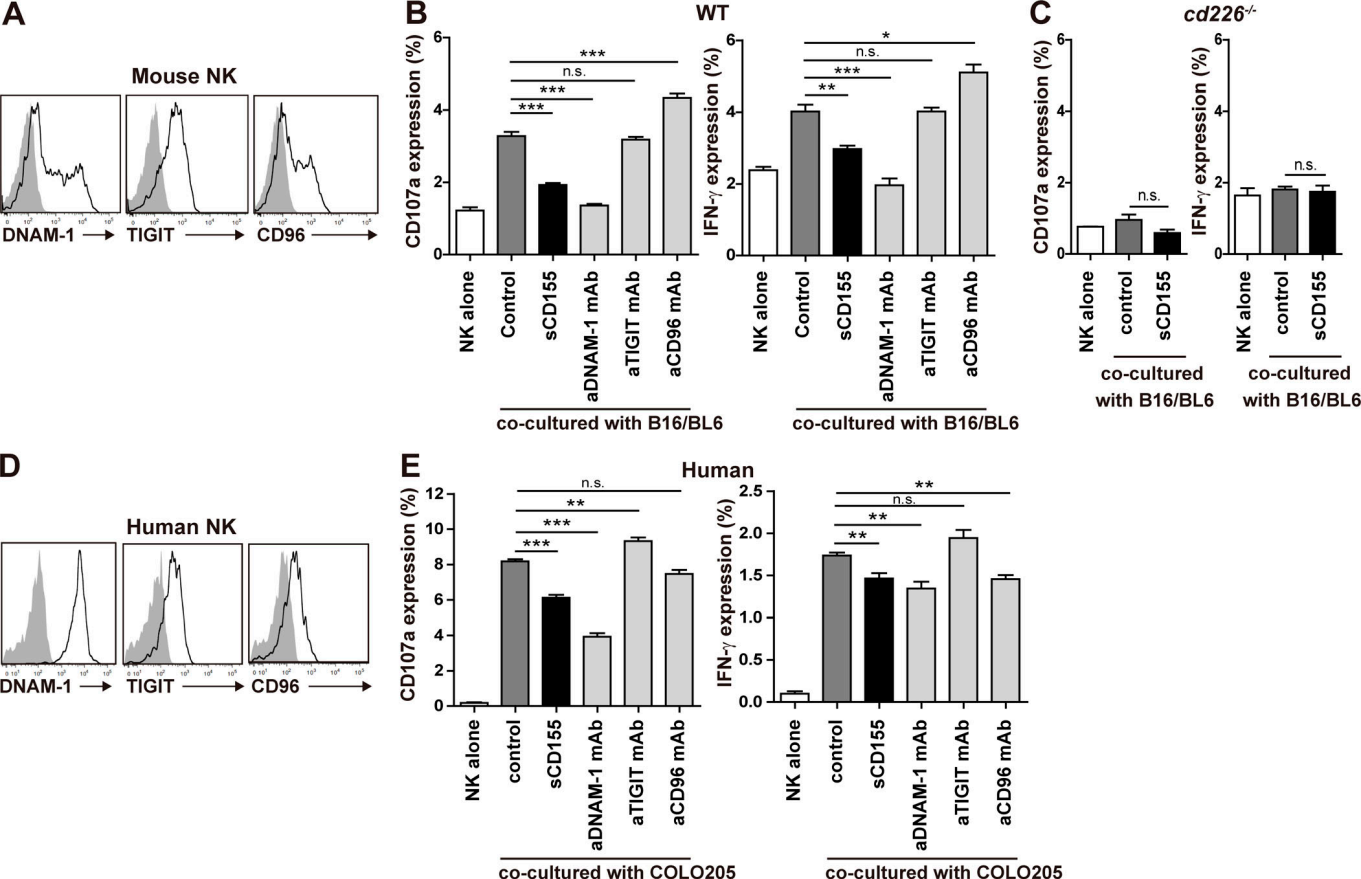
**sCD155 suppresses DNAM-1–mediated cytotoxic activity of NK cells.** **(A)** Mouse NK cells were isolated from the spleens of C57BL/6 WT mice and cultured with IL-2 for 5 d. Expression of DNAM-1, TIGIT, and CD96 on IL-2–activated mouse NK cells was analyzed by using flow cytometry. The shaded histogram indicates isotype control, and the open histogram indicates each specific antibody. **(B and C)** IL-2–activated mouse WT or *Cd226*^−/−^ NK cells were co-cultured with B16/BL6 (E:T = 1:1) in the presence of sCD155 (10 μg/ml), anti-mouse DNAM-1 mAb, anti-mouse TIGIT mAb, anti-mouse CD96 mAb, or isotype control. CD107a and IFN-γ expression was analyzed. **(D)** Human DNAM-1, TIGIT, and CD96 expression on peripheral blood NK cells isolated from a healthy donor was analyzed by using flow cytometry. Representative histograms are shown. The shaded histogram indicates isotype control, and the open histogram indicates each specific antibody. **(E)** Human NK cells were co-cultured with COLO 205 (E:T = 1:1) in the presence of sCD155 (10 μg/ml), anti-human DNAM-1 mAb, anti-human TIGIT mAb, anti-human CD96 mAb, or isotype control. CD107a and IFN-γ expression was analyzed. Error bars indicate SD. Results were analyzed by using Student’s *t* test. For all analyses: *, P < 0.05; **, P < 0.01; ***, P < 0.001; n.s., not significant.

**Figure S2.**
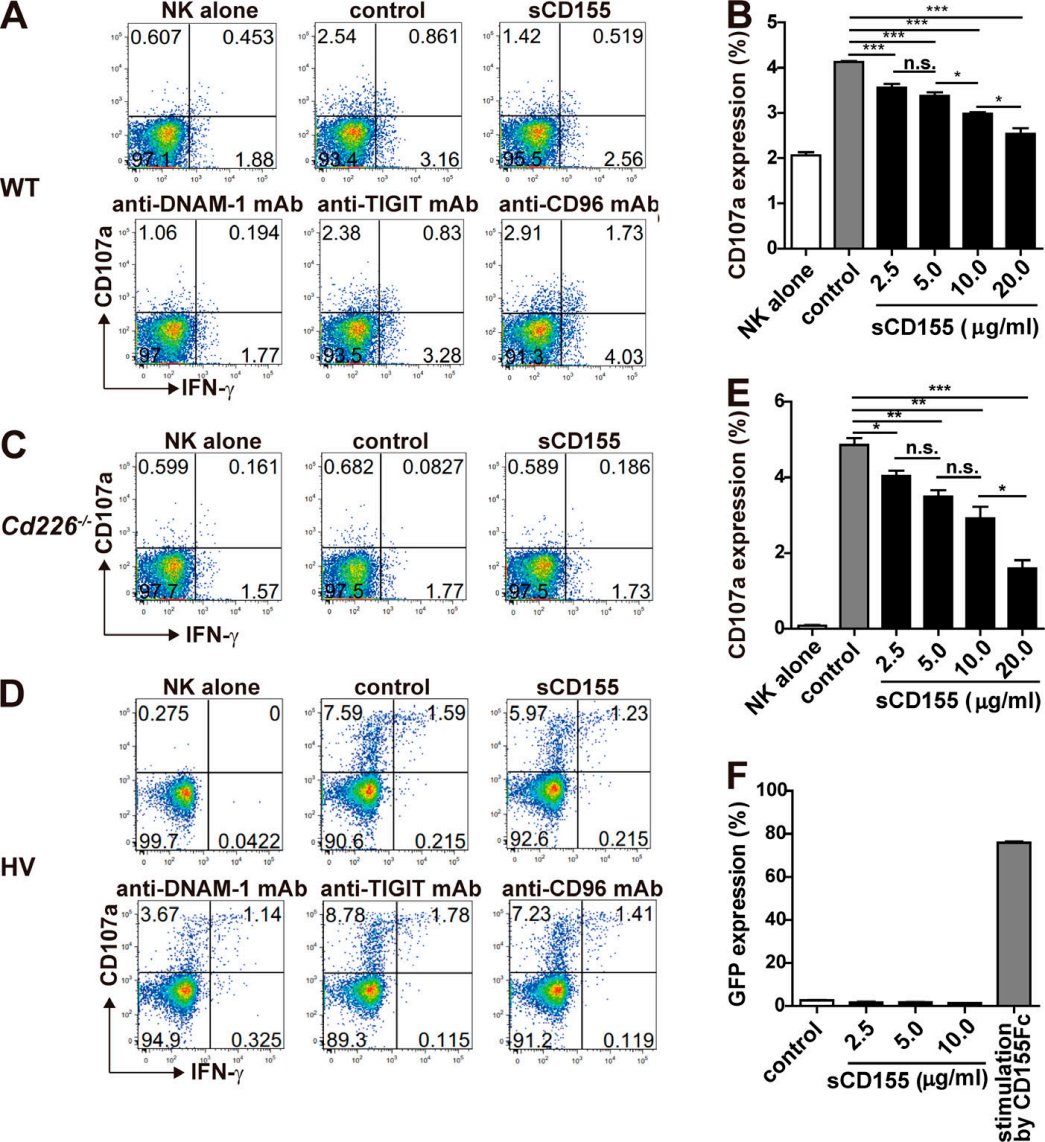
**sCD155 suppressed mouse and human NK cell activation in a DNAM-1–dependent manner.** **(A and C)** IL-2–activated mouse NK cells derived from WT (A) and *Cd226*^−/−^ (C) mice were co-cultured with B16/BL6 (E:T = 1:1) in the presence of sCD155; mAbs against mouse DNAM-1, mouse TIGIT, or mouse CD96; or isotype control and analyzed for CD107a and IFN-γ expression. **(D)** Human NK cells isolated from PBMC in a healthy volunteer (HV) were co-cultured with COLO 205 (E:T = 1:1) in the presence of sCD155; mAbs against human DNAM-1, human TIGIT, or human CD96; or isotype control and analyzed for CD107a and IFN-γ expression. **(B and E)** IL-2–activated mouse NK cells (B) and human NK cells (E) were co-cultured with B16/BL6 (E:T = 1:1) and COLO 205 (E:T = 1:1) in the presence of mouse and human sCD155, respectively, or BSA (control) and analyzed for CD107a expression. **(F)** Mouse DNAM-1–GFP reporter cells were cultured in the presence of sCD155 or BSA (control) or stimulated with plate-coated mouse CD155-Fc fusion protein and analyzed for GFP expression by flow cytometry. Data are representative of two experiments (A, C, and D). Error bars indicate SD. Results were analyzed by using Student’s *t* test. For all analyses: *, P < 0.05; **, P < 0.01; ***, P < 0.001; n.s., not significant.

To demonstrate the role of sCD155 in the degranulation activity of NK cells, we co-cultured IL-2–activated NK cells, which expressed the CD155 receptors DNAM-1, TIGIT, and CD96 (Fig. 3 A), with B16/BL6 in the presence or absence of sCD155 or neutralizing mAbs against each receptor for CD155. sCD155 significantly suppressed the degranulation of IL-2–activated NK cells, as determined by the expression of CD107α and IFN-γ, in a dose-dependent manner (Fig. 3 B and Fig. S2, A and B). Similarly, anti–DNAM-1 mAb also significantly suppressed NK cell degranulation (Fig. 3 B and Fig. S2 A). In contrast, anti-CD96 mAb significantly augmented the degranulation of NK cells, whereas anti-TIGIT mAb had no effect (Fig. 3 B and Fig. S2 A), consistent with a previous report that CD96 is a negative regulator in NK cells (Blake et al., 2016). These results are in agreement with the in vivo phenotype of the lung tumor colonization of sCD155/BL6 in mice deficient in each CD155 receptor (Fig. 2, B–D). To confirm that the suppressive effect of sCD155 on degranulation was dependent on DNAM-1, we used IL-2–activated NK cells derived from *Cd226*^−/−^ mice as effector cells. As expected, sCD155 had no effect on *Cd226*^−/−^ NK cell degranulation activity (Fig. 3 C and Fig. S2 C). These results indicated that sCD155 suppressed the DNAM-1–mediated degranulation of NK cells against B16/BL6.

We also examined whether human sCD155 suppressed DNAM-1–mediated NK cell degranulation. We isolated human NK cells from peripheral blood mononuclear cells, which expressed all of the CD155 receptors DNAM-1, TIGIT, and CD96 (Fig. 3 D), and co-cultured them with the colon cancer cell COLO 205. Although most human tumor cells, including COLO 205, express sCD155 as well as membrane-bound CD155, we speculated that the amount of endogenous sCD155 would be insufficient to modulate NK cell activation in an in vitro assay. Therefore, we added recombinant human sCD155 to the culture to observe the effect of sCD155 on NK cell degranulation. Addition of recombinant human sCD155 significantly suppressed the degranulation of human NK cells against COLO 205 in a dose-dependent manner (Fig. 3 E and Fig. S2, D and E). Similarly, a neutralizing mAb against human DNAM-1 also significantly suppressed NK cell degranulation (Fig. 3 E and Fig. S2 D). In contrast, unlike the case in mouse NK cells, anti-TIGIT mAb significantly augmented the degranulation of human NK cells, whereas anti-CD96 mAb had no effect (Fig. 3 E and Fig. S2 D), consistent with a previous report that TIGIT mediates an inhibitory signal in human NK cells (Stanietsky et al., 2009). Because DNAM-1–mediated degranulation of NK cells is dependent on membrane-bound CD155 expressed on tumor cells, sCD155 may function as a neutralizing, rather than signal-transducing, molecule for DNAM-1 on NK cells. To confirm this scenario, we established a NFAT–GFP reporter cell line expressing the extracellular and transmembrane regions of DNAM-1 fused with the intracellular region of FcRγ chain. Although the reporter cells expressed GFP upon stimulation with plate-bound chimeric protein consisting of the extracellular portion of CD155 fused with the Fc portion of human IgG (CD155-Fc), GFP was not detected in the reporter cells in the co-culture with sCD155 (Fig. S2 F). These findings suggested that sCD155 may compete with the membrane-bound CD155 for binding to DNAM-1 and likely plays a role as a ligand decoy for DNAM-1. Previous reports demonstrated that several NK cell receptors bound soluble, as well as membrane-bound, ligands, and the soluble ligands either augment or suppress NK cell–mediated tumor immunity. For example, soluble MIC and B7-H6, the ligands for NK cell–activating receptors NKG2D and NKp30, respectively, mediate the internalization of each receptor, resulting in the downmodulation of NK cell activity and suppression of tumor immunity (Groh et al., 2002; Pesce et al., 2015; Schlecker et al., 2014). In the case of sCD155, it is also possible that sCD155 induced the endocytosis of DNAM-1, resulting in the downmodulation of NK cell activity. On the contrary, soluble MULT1, a high-affinity ligand for the NK cell receptor NKG2D, suppressed the down-regulation of NKG2D expression mediated by membrane-bound MULT1 and promoted NK cell activation and tumor rejection (Deng et al., 2015).

### Affinity of sCD155 for DNAM-1 is greater than that for TIGIT and CD96

**Figure 4.**
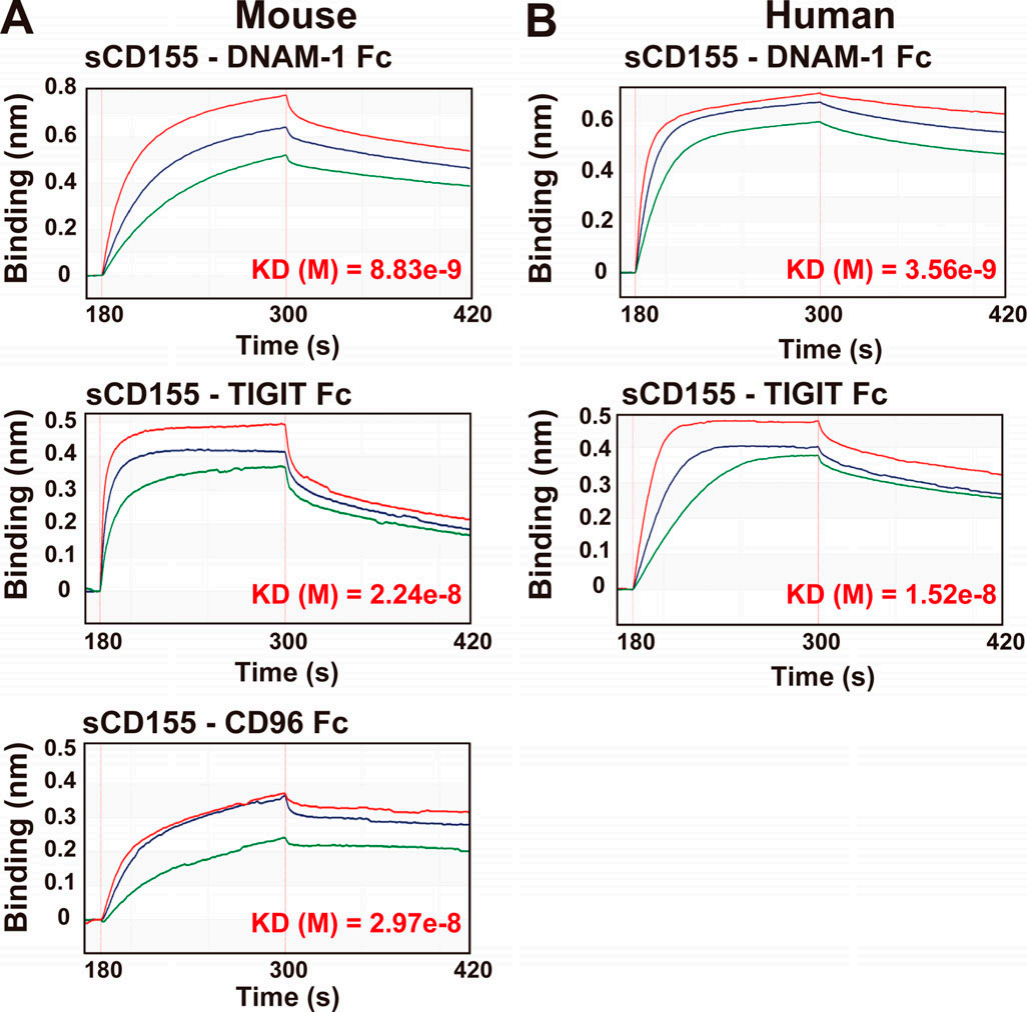
**DNAM-1 is the molecule with the greatest affinity for sCD155.** **(A)** Mouse His-tagged sCD155 was coated onto a Ni-NTA biosensor and reacted with the analytes indicated. Mouse DNAM-1–Fc fusion protein (Fc) was used at 125 nM (red), 62.5 nM (blue), or 31.25 nM (green). Mouse TIGIT-Fc was used at 307 nM (red), 153.5 nM (blue), or 76.75 nM (green). Mouse CD96-Fc was used at 762 nM (red), 381 nM (blue), or 190.5 nM (green). Antibody affinity was measured in the global analysis mode of the BLItz system. Quantified affinities (K_D_) are shown in red font. **(B)** Human His-tagged sCD155 was coated onto a Ni-NTA biosensor and reacted with the analytes indicated. Human DNAM-1–Fc was used at 500 nM (red), 250 nM (blue), or 125 nM (green). Human TIGIT-Fc was used at 615 nM (red), 307.5 nM (blue), or 153.75 nM (green). Antibody affinity was measured in the global analysis mode of the BLItz system. Quantified affinities (K_D_) are shown in red font.

We demonstrated here that the suppressive effect of sCD155 on NK cells in both mice and humans was dependent on DNAM-1, rather than TIGIT and CD96, suggesting that the binding affinity of sCD155 for DNAM-1 was greater than that for TIGIT and CD96 on these cells. To determine the binding affinity of sCD155 for each receptor, we used the extracellular portion of mouse and human CD155 tagged with His protein at the C terminus (sCD155-His) and chimeric proteins consisting of the extracellular portions of mouse and human DNAM, TIGIT, or CD96 fused with the Fc portion of human IgG (DNAM-1–Fc, TIGIT-Fc, and CD96-Fc, respectively). The affinity of sCD155-His for DNAM-1 was greater than that for TIGIT or CD96 in mice and greater than that for TIGIT in humans, as analyzed by using a biolayer interferometry technology system (Fig. 4, A and B). These results were consistent with our findings that DNAM-1, rather than TIGIT or CD96, was involved in the suppressive effect of sCD155 on NK cell cytotoxic activity in vivo and in vitro. However, previous reports have demonstrated that the binding affinity of CD155-Fc for TIGIT expressed on Chinese hamster ovary cells is greater than that for DNAM-1 expressed on these cells (Stanietsky et al., 2013; Yu et al., 2009). This discrepancy might be caused by differences in the construction of sCD155: sCD155-His forms a monomer, whereas CD155-Fc forms a dimer. Nonetheless, because sCD155 in the sera of cancer patients is a monomer (Baury et al., 2003), we consider that our results reflected the physiological function of sCD155.

**Figure S3.**
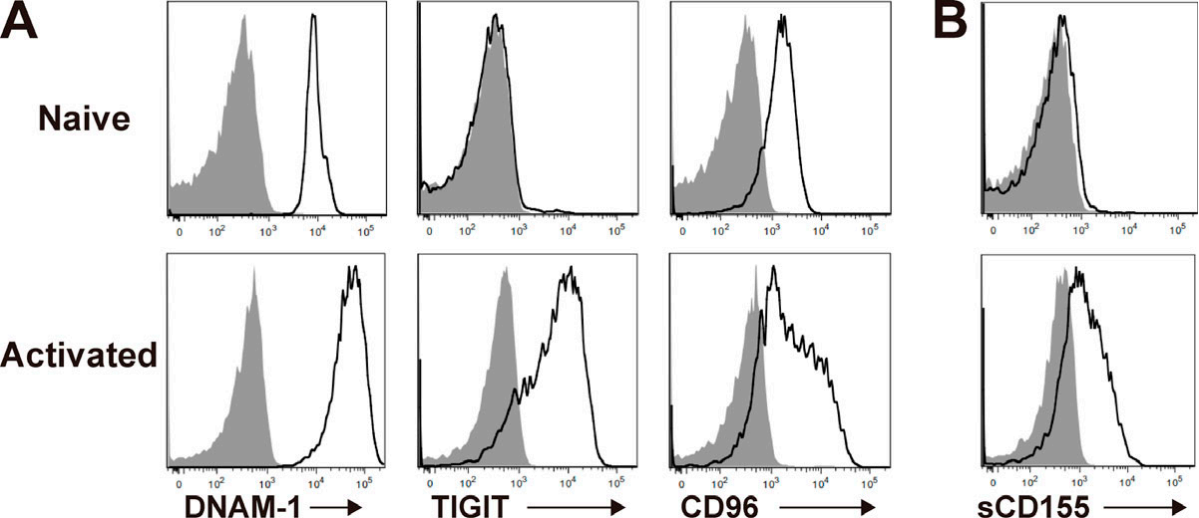
**sCD155 binds to activated CD8^+^ T cells.** **(A)** DNAM-1, TIGIT, and CD96 expression on spleen CD8^+^ T cells stimulated with or without plate-coated anti-CD3 mAb and soluble anti-CD28 mAb in the presence of IL-2 was analyzed by using flow cytometry. The shaded histogram indicates isotype control, and the open histogram indicates each specific antibody. **(B)** sCD155 binding to CD8^+^ T cells. The naive (upper) or activated (bottom) CD8^+^ T cells were incubated with recombinant mouse CD155-His and then were stained with PE-conjugated anti-His mAb. The shaded histogram indicates isotype control, and the open histogram indicates anti-His mAb staining.

Here, by using a system of experimental lung colonization of B16/BL6 melanoma in mice, we demonstrated that sCD155 interferes with the function of DNAM-1 in NK cell–mediated tumor immunity. Our results suggested that sCD155 competes with membrane-bound CD155 to bind as a ligand decoy to DNAM-1 on NK cells. Currently, it remains undetermined whether sCD155 affects not only NK cell function but also T cell function in different tumor models. Since sCD155 is also able to bind to activated T cells, which express DNAM-1, TIGIT, and CD96 (Fig. S3), sCD155 may affect T cell–mediated tumor immunity as well in certain situations.

We demonstrated previously that the expression of membrane-bound CD155 and sCD155 is up-regulated similarly in various tumor cells (Iguchi-Manaka et al., 2016). However, although it is unclear how the expression of sCD155 is regulated, certain patients show greater sCD155 expression than membrane-bound CD155 expression in their tumors (Iguchi-Manaka et al., 2016), in which cases sCD155 might lead to tumor immune evasion. Several clinical studies have shown that CD155 expression, as analyzed by immunohistochemistry, is associated with a poor prognosis in cancer patients (Bevelacqua et al., 2012; Nakai et al., 2010; Nishiwada et al., 2015). Because immunohistochemical analysis by using anti-CD155 mAb detects sCD155 as well as membrane-bound CD155, it might be sCD155, rather than membrane-bound CD155, that affects poor prognosis in these patients. In addition, a very recent study distinguished cytoplasmic CD155 from membrane-bound CD155 and demonstrated that membrane-bound CD155 expression was strongly correlated with the abundance of tumor-infiltrated NK cells. In contrast, the study reported that overall survival and disease-free survival were significantly shorter in breast cancer patients with high levels of expression of cytoplasmic CD155 than in those with low levels of expression (Triki et al., 2019). Because cytoplasmic CD155 may include sCD155, this observation is consistent with our findings in mouse models.

In recent years, TIGIT has received a lot of attention as a novel immune checkpoint molecule. The mechanisms of action of anti-TIGIT mAb are release of effector cells such as CTLs from TIGIT-mediated inhibitory signaling and augmentation of DNAM-1–CD155 interaction (Solomon and Garrido-Laguna, 2018). Although clinical trials of anti-TIGIT mAb for advanced solid cancer were initiated in 2016 (Solomon and Garrido-Laguna, 2018), we speculate that DNAM-1–mediated tumor immunity may still be diminished by sCD155 even if TIGIT is blocked. Therefore, removal of sCD155 either with or without checkpoint blockade may be a beneficial strategy. Together, our data shed light on the novel immunosuppressive function of sCD155 in the tumor microenvironment.

## Materials and methods

### Mice and cell lines

C57BL/6 WT mice were purchased from CLEA. Severely immunodeficient NOG mice (NOD/Shi-scid, IL-2Rγ deficient) were purchased from Central Institute for Experimental Animals. C57BL/6 *Cd226*^−/−^ and *Tigit*^−/−^ mice were generated as previously described (Iguchi-Manaka et al., 2008; Negishi et al., 2018). *Cd96*^−/−^ mice were generated by using a CRISPR-Cas9 system, as follows. Exon 3 of *Cd96* (5′-AAT​AGA​GAC​AAA​TCG​GAC​TC-3′) was inserted into the *pX330* vector (Addgene plasmid #42230), which was designated *pX330-Cd96*. The *pX330-Cd96* (5 ng/µl) was injected into the pronuclei of pronuclear-stage embryos of C57BL/6J female mice. Shortly afterward (15 min to 2 h), surviving pronuclear-stage embryos were transferred into the oviducts of pseudopregnant Institute of Cancer Research mice, and 123 weaned pups were obtained. These F0 mice were mated with WT C57BL/6J mice, and F1 heterozygous mice that had a seven-base deletion of *Cd96* exon 3 were obtained. These F1 heterozygous mice were mated with each other, and *Cd96*^−/−^ mice were finally established. The design of *pX330-Cd96* and the injection, followed by the transfer of embryos, were performed by the Laboratory Animal Resource Center at the University of Tsukuba. These mice were housed under specific pathogen–free conditions in the same room at the Animal Resource Center at the University of Tsukuba. All animal experiments were approved by the Institutional Review Committee and applied according to the guidelines of the University of Tsukuba. The B16/BL6 melanoma cell line, the COLO 205 colon cancer cell line, and the cervix adenocarcinoma cell line HeLa were cultured in RPMI-1640 (Sigma-Aldrich) containing 10% FBS, 10 mM Hepes, 1 mM sodium pyruvate, MEM Non-Essential Amino Acids Solution (1/100 vol/vol), and L-glutamine penicillin streptomycin solution (1/100 vol/vol; Thermo Fisher Scientific).

### Generation of mouse sCD155 expressing B16/BL6

The sequence of CD155 exons 1–5 was incorporated into the vector p3xFLAG-CMV-13. Consequently, exons 6–8 of the *Cd155* gene were replaced with DYKDDDDK to generate mouse sCD155-flag (sCD155). The plasmid generated was transduced into the B16/BL6 mouse melanoma cell line by using Lipofectamine 2000 (Thermo Fisher Scientific), and the cells were selected for resistance to G418 antibiotic (Sigma-Aldrich) and single-cell-sorted by FACS Aria (BD Bioscience). To select one clone with high production of sCD155-FLAG, FLAG-tagged protein in the culture supernatant was detected by using the sandwich ELISA system. A plate was coated with anti-mouse CD155 mAb (TX56, previously generated; Iguchi-Manaka et al., 2008); the sample supernatant was then applied, followed by biotinylated anti-FLAG (M2) and HRP-conjugated streptavidin. The selected cells were termed “sCD155/BL6.” Empty vector–transduced B16/BL6 was prepared in the same way and was termed “mock/BL6.”

### Measurement of sCD155

Human sCD155 concentrations in the culture supernatants of HeLa (2.0 × 10^4^ cells/100 µl/well) 24 h after the start of culture were measured by ELISA, as described (Iguchi-Manaka et al., 2016). For measurement of mouse sCD155, we established a cytokine bead array (CBA) assay. Anti-CD155 mAb (TX56) was conjugated to BD CBA Functional Beads (BD Bioscience) in accordance with the manufacturer’s protocol. 2 μl of the TX56 beads was incubated with a 50-µl sample of sera, culture supernatants, or tumor extraction at room temperature or on ice for 2 h, washed with the wash buffer (BD Bioscience), and then incubated with 0.5 µg/ml of rabbit anti-mouse CD155 polyclonal antibody generated in our laboratory in 50 µl of the Assay Diluent (BD Bioscience) at room temperature for 1 h. The TX56 beads were then washed with the wash buffer, incubated with 1 µl of PE-conjugated donkey anti-rabbit polyclonal IgG (BioLegend) in 50 µl of the Assay Diluent at room temperature for 1 h, washed again with the wash buffer, and analyzed by flow cytometry. The culture supernatants of sCD155/BL6 and mock/BL6 (2.0 × 10^4^ cells/100 µl medium/well) obtained 24 h after the start of culture, sera of peripheral blood of mice injected with sCD155/BL6 or mock/BL6, and sera from the pulmonary vein of tumor-colonized lungs after clamping were subjected to the CBA assay.

### In vivo lung colonization models

C57BL/6 WT, *Cd226*^−/−^, *Tigit*^−/−^, *Cd96*^−/−^, and *Rag-1*^−/−^ mice (8–10 wk old, males) raised under the same environment were intravenously inoculated with sCD155/BL6 or mock/BL6 cells at 1.0 × 10^5^/100 µl in cold PBS. After 17 d (17 and 21 d in the case of *cd96*^−/−^), the lungs were resected after perfusion with PBS. Lung colonization was quantified by counting the visible colonies on the lung surface under a stereo microscope. Anti-mouse NK1.1 mAb (100 µg/mouse; PK136) or anti-mouse CD8 mAb (200 µg/mouse; 53–6.7) was injected intraperitoneally into C57BL/6 WT mice on days 4, 3, and 10 (tumor cells were injected on day 0) to deplete NK cells and CD8^+^ T cells, respectively. To confirm the efficacy of depletion, peripheral blood mononuclear cells were stained with anti-CD3ε-FITC (145-2C11; Tonbo), anti-CD49b-PE (DX5; BD Biosciences), and anti-CD8-PE (53–6.7; BD Biosciences). In all experiments, the numbers of B16/BL6 colonies in the lung were counted blindly without any information on each mouse to exclude bias.

### Ligand expression on NK cells in B16/BL6 colony in lung

C57BL/6 WT mice were inoculated intravenously with B16/BL6 (1.0 × 10^5^ cells/100 µl). After 17 d, colonies in the lung were carefully taken from the PBS-perfused lung under a stereo microscope and were incubated at 37°C for 1 h in RPMI medium (Sigma-Aldrich) containing DNase I (100 U/ml). The foci were mashed and passed through a mesh to prepare a single-cell suspension. Isolated cells, including tumor cells, were stained with biotinylated anti-mouse DNAM-1 followed by streptavidin-PE (BD Biosciences), anti-mouse TIGIT-PE (1G9; Tonbo), and anti-mouse CD96-PE (630612; R&D Systems) under staining with anti-CD45.2-PerCP-Cy5.5 (Ly-5.2; BD Biosciences), anti-CD3-APC (145-2C11; BD Biosciences), and anti-NK1.1-FITC (PK136; BD Bioscience) to gate on NK cells.

### Opal multiplex immunofluorescence staining

Lungs taken from C57BL/6 mice 17 d after intravenous inoculation of sCD155/BL6 or mock/BL6 (1.0 × 10^5^ cells/100 µl) were formalin fixed and paraffin embedded, and the resulting tissue blocks were cut into 7-µm sections and placed on glass slides. The sections on the slides were dried on a hot plate and then rinsed with 100% xylene for 10 min three times and deparaffinized with a graded series of ethanol for 5 min per grade. The slides were rinsed in 0.3% H_2_O_2_–MeOH at room temperature for 30 min to block endogenous peroxidase. Antigens were retrieved with AR6 buffer in microwave treatment at 700 W for 2 min, followed by heating in the same buffer on a hot plate at 95–100°C for 15 min. Blocking was performed with blocking buffer (10× casein solution; Vector Laboratories) overnight at 4°C. Blocking buffer containing the primary antibody against NKp46 (1/200, polyclonal; Abcam) was placed on the section on each slide and left overnight at 4°C. The slides were then washed and incubated with the secondary antibody, anti-rabbit IgG–HRP (1/300, polyclonal; BioLegend), at room temperature for 1 h. NK cells were visualized by incubation with Opal 570 reagent (Perkin Elmer) at room temperature for 10 min. Afterwards, to stain for sCD155-FLAG produced by sCD155/BL6, the slides were again treated for antigen retrieval as described above. Antibody diluent/blocker (Perkin Elmer) containing anti-FLAG antibody (1/50, D6W5B; Cell Signaling Technology) was placed on the section overnight at 4°C. The slides were then washed and incubated with secondary anti-rabbit IgG-HRP (1/300) at room temperature for 1 h. FLAG-positive cells were visualized by incubation with Opal 520 reagent (Perkin Elmer) at room temperature for 10 min. Finally, nuclei were stained with Vectashield mounting medium with DAPI (Vector Laboratories), and the section was covered by a glass cover with Prolong Diamond Antifade Mountant (Invitrogen). Ten parts of the stained sections were selected and photographed randomly. At least 30 NKp46^+^ cells from each lung of sCD155/BL6- or mock/BL6-inoculated mice were observed under the Mantra Quantitative Pathology Workstation (Perkin Elmer). The frequency of FLAG^+^ cells among NKp46^+^ cells was quantified.

### Mouse NK cell degranulation assay

Mouse NK cells were sorted by negative selection with Dynabeads MyOne Streptavidin C1 (Thermo Fisher Scientific) and biotinylated anti-mouse CD4 (H129.19), CD8 (53–6.7), CD19 (1D3), CD5 (53–7.3), Gr-1 (RB6-6C5), and Ter119 (TER119) antibodies (BD Biosciences) from whole splenocytes prepared from naive C57BL/6 WT mice. Isolated NK cells were cultured in 24-well plates at 37°C for 120 h in RPMI 1640 containing 10% FBS, 10 mM Hepes, 1 mM sodium pyruvate, MEM Non-Essential Amino Acids Solution (1/100 vol/vol), L-glutamine penicillin streptomycin solution (1/100 vol/vol), and 50 µM 2-mercaptoethanol in the presence of 100 ng/ml IL-2 (BD Biosciences). Afterwards, activated NK cells were incubated on ice for 20 min with ∼2.5–20 µg/ml of recombinant mouse CD155 protein (monomeric structure; R&D Systems); anti-mouse DNAM-1 (TX42), TIGIT (TX99), or CD96 monoclonal antibody (6A6; eBioscience); or isotype rat IgG2a (BD Biosciences) as a control. The pretreated NK cells were co-cultured with B16/BL6 (effector:target [E:T] = 1:1) in the presence of Protein Transport Inhibitor (1/4,000; BD Biosciences) and APC-conjugated anti-mouse CD107a (1/200, 1D4B; BioLegend) at 200 µl/well in 96-well round-bottomed plates at 37°C in a 5% CO_2_ incubator for 5.5 h. Co-cultured cells were stained with PerCP-Cy5.5 conjugated anti-mouse NK1.1 (PK136; BioLegend) and FITC-conjugated anti-mouse CD3 (145-2C11; BD Biosciences) and CD19 (1D3; BD Biosciences) to gate on NK cells in the flow cytometry analysis. IFN-γ was detected by intracellular staining with PE-conjugated anti-mouse IFN-γ (XMG1.2; BD Biosciences).

### Human NK cell degranulation assay

Magnetic-activated cell-sorting positive selection with CD56 microbeads (Miltenyi Biotec) was used to isolate human NK cells from peripheral blood mononuclear cells collected from healthy volunteers. Isolated NK cells were incubated on ice for 20 min with ∼2.5–20 µg/ml of recombinant human CD155 protein (monomeric structure; R&D Systems); anti-human DNAM-1 (TX94), TIGIT (MBSA43; Invitrogen), or CD96 monoclonal antibody (NK92.39; BioLegend); or isotype mouse IgG1 (MOPC-21; BioLegend) as a control. Pre-treated NK cells were co-cultured at 37°C in a 5% CO_2_ incubator for 5.5 h with COLO 205 (E:T = 1:1) in the presence of Protein Transport Inhibitor (1:2,000; BD Bioscience) and APC-conjugated anti-human CD107a (1/200, H4A3; BioLegend) in 96-well round-bottomed plates. Co-cultured cells were stained with V450-conjugated anti-human CD56 (B159; BD Biosciences) and FITC-conjugated anti-human CD3 (UCHT1; Tonbo) and CD19 (HIB19; BD Biosciences) to gate on NK cells in the flow cytometry analysis. IFN-γ was detected by intracellular staining with PE-conjugated anti-human IFN-γ (4S.B3; BD Biosciences).

This study was approved by the ethical review boards of the University of Tsukuba (no. 234–1).

### Mouse DNAM-1 reporter assay

The NFAT–GFP reporter BaF/3 cell line provided by H. Arase (Osaka University, Osaka, Japan) was transfected with cDNA encoding the chimeric protein consisting of the extracellular and transmembrane regions of DNAM-1 fused with the intracellular region of FcRγ chain (DNAM-1-Fc). The reporter cells were incubated with 10 µg/ml of BSA or 2.5, 5.0, or 10 µg/ml of recombinant mouse CD155 protein (R&D Systems) at 37°C for 18 h. For positive control of GFP expression, plate-coated CD155-Fc protein (5 µg/ml) was used. GFP expression of the reporter cells was analyzed by flow cytometry.

### Affinity assay

For studies in mice, an Ni-nitrilotriacetic acid (Ni-NTA) biosensor (Molecular Devices) was coated with His-tagged recombinant mouse CD155 (20 µg/ml; R&D Systems). Mouse DNAM-1 Fc fusion protein (125 nM, 62.5 nM, and 31.25 nM), TIGIT Fc fusion protein (307 nM, 153.5 nM, and 76.75 nM), and CD96 Fc fusion protein (762 nM, 381 nM, and 190.5 nM) were used as analytes for association with coated His-tagged recombinant mouse CD155. Each mouse Fc fusion protein was generated by using an HEK 293T expression system. For human studies, the Ni-NTA biosensor was coated with His-tagged recombinant human CD155 (20 µg/ml; R&D Systems). Human DNAM-1 Fc fusion protein (500 nM, 250 nM, and 125 nM), TIGIT Fc fusion protein (615 nM, 307.5 nM, and 153.75 nM), and CD96 Fc fusion protein (762 nM, 381 nM, and 190.5 nM) were used as analytes for association. Each human Fc fusion protein was purchased from R&D Systems. All samples were diluted with kinetics buffer (Molecular Devices). All doses of each analyte were selected in accordance with the condition that the *R*^2^ value was over 0.98. The times for the set of analysis steps are shown in Table 1. Quantifications were done by using the global mode of BLItz (Molecular Devices).

**Table 1.** Steps used in the BLItz system affinity assay

Step type	Duration (s)	Solution
Initial baseline	30	Kinetics buffer
Loading	120	His-tagged recombinant CD155
Baseline	30	Kinetics buffer
Association	120	Fc fusion protein
Dissociation	120	Kinetics buffer

### Online supplemental material

Fig. S1 shows the characterization of generated sCD155/BL6 and mock/BL6 and serum levels of sCD155 in B16/BL6 lung-colonization models. Fig. S2 shows dot plots of flow cytometry analysis in the NK cell degranulation assay and the mouse DNAM-1-GFP reporter assay. Fig. S3 shows the binding of sCD155 to naive and activated CD8^+^ T cells.
